# The Effect of Occupational Lifting on Hypertension Risk: Protocol for a Project Using Data From the Copenhagen City Heart Study

**DOI:** 10.2196/resprot.9692

**Published:** 2018-04-27

**Authors:** Mette Korshøj, Harald Hannerz, Jacob Louis Marott, Peter Schnohr, Eva Irene Bossano Prescott, Els Clays, Andreas Holtermann

**Affiliations:** ^1^ National Research Centre for the Working Environment Copenhagen Denmark; ^2^ Copenhagen City Heart Study Frederiksberg and Bispebjerg Hospital University of Copenhagen Copenhagen Denmark; ^3^ Department of Public Health Ghent University Ghent Belgium; ^4^ Department of Sports Science and Clinical Biomechanics University of Southern Denmark Odense Denmark

**Keywords:** occupational exposure, blood pressure, Copenhagen City Heart Study, cardiovascular diseases, manual handling, blue collar, occupational epidemiology, heavy lifting, cohort study

## Abstract

**Background:**

Hypertension is a major risk factor for cardiovascular disease and is responsible for 14% of all annual deaths globally. The prevalence of hypertension varies across occupational groups, possibly affected by differences in the working environment. One work-related factor that might impose a risk for hypertension is lifting due to the acute large increases in blood pressure (BP) during lifting.

**Objective:**

The aim of this study is to explore associations between heavy occupational lifting and hypertension in the Copenhagen City Heart Study.

**Methods:**

This study will use data from the third, fourth, and fifth examination of the Copenhagen City Heart Study. The dataset contains person-based information on health as well as a large variety of biological, environmental, and lifestyle-related factors. Using a cross-sectional design, we will investigate the association between heavy occupational lifting and hypertension, defined as using antihypertensive drugs or having a measured systolic BP (SBP) ≥140 mm Hg or diastolic BP (DBP) ≥90 mm Hg. Furthermore, in a prospective design, we will investigate the association between heavy occupational lifting and risk of becoming an SBP case, defined as the shift from not using antihypertensive drugs in examination n to use of antihypertensive drugs in examination n+1 or an above median delta value of SBP (SBP in examination n+1−SBP in examination n).

**Results:**

In the third examination in 1991-1994, 10,135 out of 16,560 participants attended (61.20%), in the fourth examination in 2001-2003, 6237 out of 12,599 participants attended (49.50%), and in the fifth examination in 2011-2015, 4550 out of 9765 participants attended (46.59%). On the basis of the inclusion criteria of answering to the level of occupational physical activity, 5031 observations were excluded from examination 3, 2600 from examination 4, and 1621 from examination 5. Hence, the final populations for the cross-sectional and prospective analysis are assumed to include less than 7166 participants in the cross-sectional analysis and less than 1850 participants in the prospective analysis due to the additional inclusion criteria of measured BP and use of antihypertensive drugs.

**Conclusions:**

One-third of the workforce in Europe reports to carry or move heavy loads regularly during working hours (6th survey in Eurofound). Thus, if this study shows occupational lifting to increase the risk for hypertension, the prevention for hypertension can be improved.

## Introduction

### Background

Hypertension is a major risk factor for cardiovascular diseases [1,2] and is responsible for 14% of all annual deaths globally [1]. The prevalence of hypertension varies across occupational groups, possibly affected by differences in the working environment. One work-related factor that might impose a risk for hypertension is lifting [3,4]. Heavy lifting causes acute large increases in blood pressure (BP) [5]. These increases in BP during heavy lifting are explained by the constriction of the vessels due to contraction of muscle fibers surrounding the vessels as well as the pressor reflex, both leading to an increased peripheral resistance and thereby also an increased BP [5]. Thus, because some workers perform occupational lifting for several hours per day, many days per week, higher BP or hypertension is likely to occur [6]. Yet, scientific knowledge of the relation between heavy occupational lifting and hypertension is limited. Previous studies investigating this relation have found occupational lifting to increase risks for myocardial infarction [3] and ischemic heart disease [4] in population studies including both sexes and workers from white- and blue-collar occupations. However, one study, only including males from blue-collar occupations did not find increased risk for ischemic heart disease from occupational lifting [7]. As the study by Petersen and colleagues [4] found the risks from lifting to be most pronounced among workers with low occupational physical activity (OPA) but high exposure to lifting, it seems that investigations of associations between occupational lifting and risk for hypertension benefit from populations including both sexes and a variety of occupations.

Conversely, heavy lifting might also impose beneficial effects on BP, since resistance training involving heavy lifting has been shown to reduce resting BP [8,9]. Additionally, it is also unknown whether effects of exposure to heavy occupational lifting differ between participants with and without preexisting hypertension. A Danish survey from 2016 [10] concludes that 22% of the Danish workforce are exposed to occupational lifting during ≥25% of their working hours. Likewise, 32% of European workers report to carry or move heavy loads regularly during working hours (6th survey in Eurofound). Thus, an investigation of the association between occupational lifting and risk of hypertension in population studies including both sexes and both blue- and white-collar occupations, might uncover a potential for prevention of cardiovascular diseases for a quite large proportion of the working population.

### Objective

The aim of this study is to explore associations between heavy occupational lifting and hypertension in the Copenhagen City Heart Study. Associations will be investigated both cross-sectionally and prospectively, among randomly selected citizens from two districts of Copenhagen, Denmark.

For the cross-sectional analysis, the primary null-hypothesis is that there is no association between heavy occupational lifting and hypertension. For the prospective analysis, the primary null-hypothesis is that there is no association between heavy occupational lifting at baseline and increased resting systolic BP (SBP) 10 years later.

## Methods

### Overview

This study will use data from the Copenhagen City Heart Study, which have been collected via health examinations and questionnaires in five examinations, namely 1976-1978, 1981-1983, 1991-1994, 2001-2003, and 2011-2014, on random population samples from two districts of Copenhagen. The sample of the first examination consisted of approximately 20,000 people in the age range of 20 to 93 years. The samples of the other examinations consisted of all previously invited people plus a new sample of people, who were younger than 20 years at the time of the first examination. In the first examination, 73.58% responded (14,223/19,329), this dropped to 49.50% (6237/12,599) in the fourth examination [11]. The dataset contains person-based information on health, as well as a large variety of biological, environmental, and lifestyle-related factors. This study will include data from the third, fourth, and fifth examination of the Copenhagen City Heart Study for the analysis of the association between heavy occupational lifting and hypertension without inclusion of effect of time. Using a cross-sectional design, we will investigate the association between heavy occupational lifting and hypertension, defined as using antihypertensive drugs or having a measured SBP ≥140 mm Hg or DBP ≥90 mm Hg. Furthermore, in a prospective design, we will investigate the association between heavy occupational lifting and risk of becoming an SBP case across a time span of approximately 10 years. An SBP case will be defined as the shift from not using antihypertensive drugs in examination n to use of antihypertensive drugs in examination n+1 or an above median delta value of SBP (SBP in examination n+1−SBP in examination n). Analyses of associations both cross-sectional and prospectively hold the potential of evaluating associations both with and without inclusion of the effect of time.

### Inclusion Criteria

For the cross-sectional analysis, the criteria for inclusion will be participation in the BP measurement and having responded to the questions regarding level of OPA (also including heavy lifting) and antihypertensive drug usage.

Inclusion criteria for the prospective analysis will be (1) that the participant answered the question regarding level of OPA at the third examination and/or fourth examination (n); (2) that he or she was normotensive at examination n; and (3) that he or she participated in the BP measurement and gave a valid answer to the questions regarding antihypertensive drug usage in examination n and n+1.

We believe that potential effects of heavy occupational lifting on BP may be concealed, reversed, or otherwise distorted by effects from antihypertensive drugs. The reason for excluding participants with hypertension at baseline from the prospective analysis is that they either are treated with antihypertensive drugs at examination n or, due to being detected as hypertensive at the health examination, are likely to receive treatment with antihypertensive drugs in the time period between examination n and examination n+1.

### Assessment of Exposure

In all 3 examinations, the self-reported information on level of OPA was obtained by asking the question: “Please describe your level of OPA within the past year” with the following response categories: “(1) predominantly sedentary; (2) sitting or standing, some walking; (3) walking, some handling of material; (4) heavy manual work.” If answering 3 or 4, an additional question regarding heavy occupational lifting was applied. The question was: “Do you lift heavy burdens?” with the response categories: “(1) yes and (2) no.” Participants will be classified as exposed to heavy occupational lifting by answering “yes” to the question concerning heavy burdens, and those participants answering 1, 2, and 3 or 4 in combination with not lifting heavy burdens will be classified as the reference group.

Between the examinations of data collection, we do not have any information about their exposure to OPA or lifting. However, for the prospective analysis, a measure of the stability of exposure was accounted for by cross-tabulating the self-reported exposure at examination 3 by exposure at examination 4 and also the self-reported exposure at examination 4 by exposure at examination 5. Among those participants responding to the self-reported exposure to OPA at examinations 3 and 4, 13.4% (329/2459) stated to be exposed to heavy lifting in examination 3 and 12.0% (295/2459) in examination 4. Among those participants responding to the self-reported exposure to OPA at examinations 4 and 5, 8.29% (146/1762) stated to be exposed to heavy lifting in examination 4 and 6.81% (120/1762) in examination 5. An evaluation of the agreement (Cohen kappa) between exposure to heavy occupational lifting in examinations 3 and 4 was .30, and the agreement between exposure to heavy occupational lifting in examinations 4 and 5 was .40, indicating a fair agreement between exposure to heavy occupational lifting across examinations (see Tables 1-4) [12].

**Table 1:** Number of participants who responded to the questions on level of occupational physical activity (OPA) at examinations 3 and 4.

Examination 3 (1991-1994)	Examination 4 (2001-2003)			
	1^a^	2	3	4
1	599	223	45	9
2	173	511	123	8
3	57	210	367	34
4	3	21	26	50

^a^1=predominantly sedentary; 2=sitting or standing, some walking; 3=walking, some handling of material; 4=heavy manual work.

**Table 2:** Number of participants who reported to have heavy occupational lifting at examinations 3 and 4.

Examination 3 (1991-1994)	Examination 4 (2001-2003)		
	Yes	No
Yes	236	93
No	59	90

**Table 3:** Number of participants who responded to the questions on level of occupational physical activity (OPA) at examinations 4 and 5.

Examination 4 (2001-2003)	Examination 5 (2011-2015)			
	1^a^	2	3	4
1	523	163	30	1
2	175	352	73	3
3	43	112	219	13
4	7	13	13	22

^a^1=predominantly sedentary; 2=sitting or standing, some walking; 3=walking, some handling of material; 4=heavy manual work.

**Table 4:** Number of participants who reported to have heavy occupational lifting at examinations 4 and 5.

Examination 4 (2001-2003)	Examination 5 (2011-2015)	
	Yes	No
Yes	94	52
No	26	95

### Assessment of Outcome

The primary outcome in the cross-sectional analysis will be hypertensive status. Participants will be classified as hypertensives if they use antihypertensive drugs or they had a measured SBP ≥140 mm Hg or DBP ≥ 90 mm Hg.

In the prospective analysis, the primary outcome will be classified as an SBP case. The SBP case definition is the shift from no use of antihypertensive drugs in examination n to use of antihypertensive drugs in examination n+1 or an above median delta value of SBP (SBP in examination n+1−SBP in examination n).

In addition, secondary analyses will be conducted with pulse pressure (pulse pressure=SBP−DBP), mean arterial pressure (mean arterial pressure=([2 × DBP] + SBP/3) and mid BP (½ SBP + ½ DBP) as outcomes [13].

### Assessment of Covariates

Previously a number of factors have been shown to be associated both with occupational workload and BP. Thus, those factors will be included as covariates: sex (male or female) [14,15]; age (categories of <40, 50-59, 60-69, 70-79, and >80 years) [16]; body mass index (BMI; categories of <18.5, 18.5-24.9, 25.0-29.9, and ≥30kg/m^2^) [17,18] calculated from objectively measured body height and weight; smoking (categories of nonsmoking and currently smoking) [19,20]; length of education (categories of uneducated, low educated up to 3 years, vocationally educated 1-3 years, higher educated, and academically educated) [1,21]; for the prospective analysis only, additional adjustment for vital exhaustion, split in 4 categories defined elsewhere (0, 1-4, 5-9, and 10-17) [22,23]; self-rated cardiorespiratory fitness (categories of lower, similar, and higher cardiorespiratory fitness compared with peers of same sex and age) [24]; SBP at baseline (categories of 80-89, 90-99, 100-109, 110-119, 120-129, 130-139, and ≥140 mm Hg) [13]; and DBP at baseline (categories of 40-49, 50-59, 60-69, 70-79, 80-89, and ≥90 mm Hg).

### Criteria for Statistical Significance

The overall significance level will be set at .05. A Bonferroni correction will be applied, due to the similarity of the two proposed hypotheses, which means that each of the two primary hypotheses will be tested at a significance level of *P*=.025. Secondary analyses will be regarded as exploratory and will therefore not be tested for statistical significance, but the precision will be reported by 95% CI. They may influence the interpretation of findings of the primary analyses.

### Primary Statistical Analyses

Logistic regression will be used to estimate the odds of becoming a case from examination n to n+1 as a function of heavy occupational lifting. For the cross-sectional analysis, there will be a possibility of 3 observations per participant, 1 from each examination. For the prospective analysis, there will be a possibility of 2 observations per participant, 1 from the third to the fourth examination and one from the fourth to the fifth examination. The cross-sectional analysis will be controlled for sex, age, BMI, smoking, and education. The prospective analysis will, in addition to the variables of the cross-sectional analysis, be controlled for self-rated cardiorespiratory fitness, vital exhaustion, and BP at baseline. Self-rated cardiorespiratory fitness and vital exhaustion will only be included as covariates in the prospective analysis where the main point of interest is new cases and not prevalent cases as in the cross-sectional analysis. Generalized estimating equations will be used to estimate the parameters. Observations from the same person will be treated as repeated measurements. A first order autoregressive correlation structure is assumed. Should the estimated covariance matrix fail to converge, then we will resort to a variance component correlation structure.

The significance test will be based on the empirical SE and the Wald Statistic. The odds ratio (OR) between the exposed and the nonexposed will be calculated and presented with a 95% CI. The CI will be based on the empiric SE.

### Statistical Power

The power calculations are based on, inter alia, the following assumptions:

- In total, 20% of the participants were hypertensive at baseline [16].
- In total, 15.68% (1830/11,670) participants performed heavy occupational lifting at baseline (Table 2).
- In total, 55% of the participants who were normotensive at examination n would meet the case criteria (antihypertensive drug usage or an above median delta of SBP) at examination n+1.
- The intraperson correlation coefficient equals .5 in the cross-sectional analysis and .1 in the prospective analysis.

Table 5 shows the expected numbers of observations, participants, and “cases” that will be included in the primary analyses. It also shows the variance inflation factor, which is a function of the assumed intraperson correlation and the mean numbers of observations per participant.

**Table 5:** Number of observations, participants, and estimated cases that we expect to include in the primary analyses.

Analysis	Number of observations	Number of participants	Observations/Participants	Estimated number of cases^a^	Variance inflation factor
Cross-sectional	11,670	7166	1.63	2334	1.31
Prospective	4746	3271	1.45	2610	1.05

^a^A case in the primary analysis will be defined as the shift from no use of antihypertensive drugs in examination n to use of antihypertensive drugs in examination n+1 or an above median delta value of systolic blood pressure (systolic blood pressure in examination n+1-systolic blood pressure in examination n).

**Figure 1:**
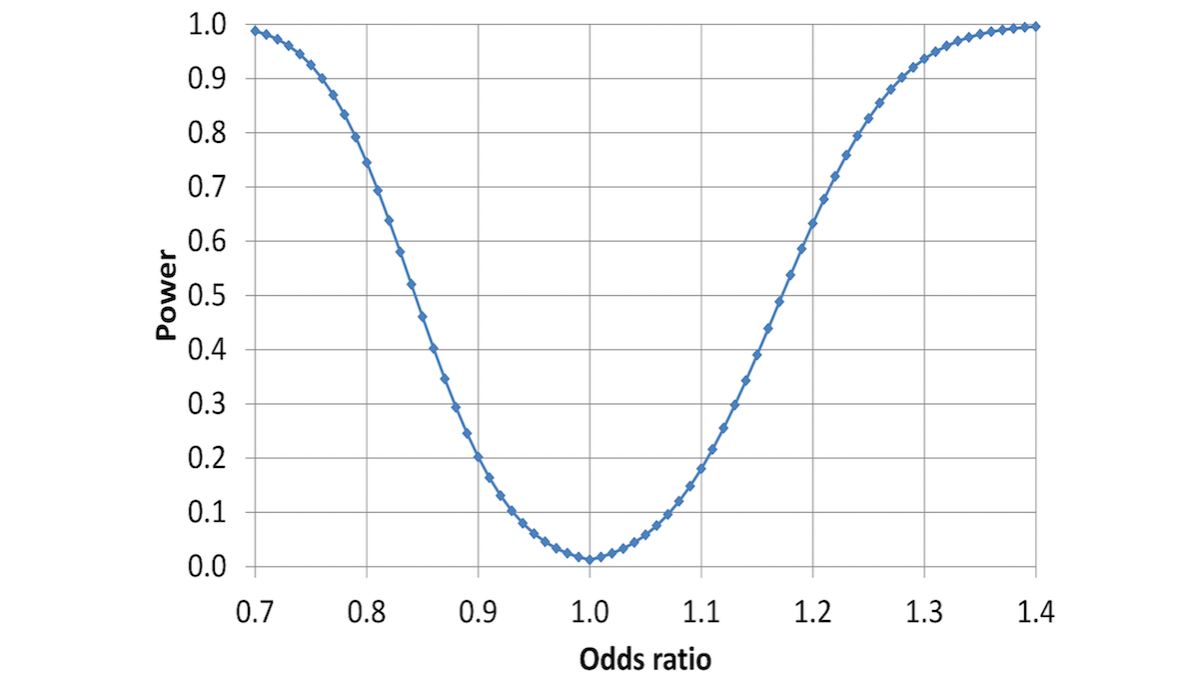
Statistical power of detecting a cross-sectional association between heavy occupational lifting and hypertension, as a function of the underlying odds ratio between exposed and unexposed participants in the target population.

**Figure 2:**
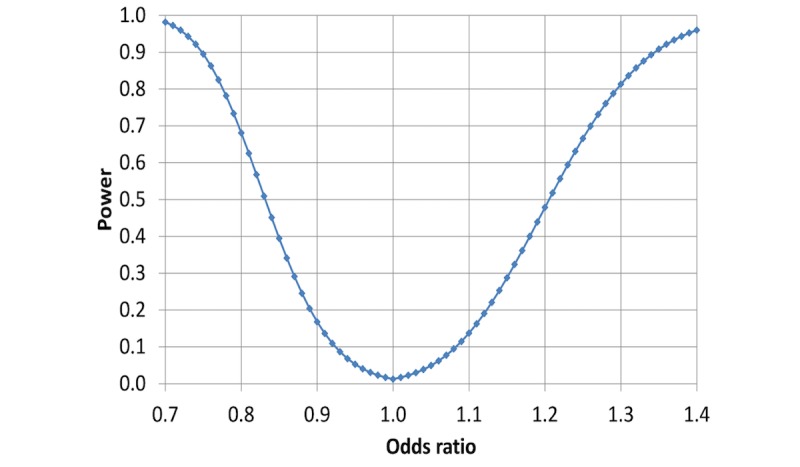
Statistical power of detecting an association between heavy occupational lifting at examination n and antihypertensive drug usage or an above median delta of systolic blood pressure (SBP) at examination n+1, as a function of the underlying odds ratio between exposed and unexposed participants in the target population.

The statistical powers of the primary hypotheses are given in Figures 1 and 2. The calculations are based on the above assumptions, the propagation of error formulas, the central limit theorem, and a two-tailed significance level at *P*=.025, for each of the two hypotheses.

### Secondary Analyses

#### Linear Regression on Systolic Blood Pressure

It has been suggested that each mm Hg increase in resting SPB is associated with an approximately 3.5% increased risk of death due to ischemic heart disease (IHD) [13]. It has moreover been suggested that the relative effect of a 1 mm Hg increase is quite independent of the level of SBP; a change in SBP from 120 to 121 would, for example, cause the same relative risk increase as a change from 139 to 140 [13]. From this viewpoint, it would be of interest to estimate the expected effect of heavy occupational lifting on resting SBP in a linear regression model and thereby obtain an estimate that could be directly translated into relative risks of death due to IHD. There are, however, some problems with this approach:

If occupational lifting is associated with risk of hypertension and we exclude participants who are treated for hypertension, then the participants who had been most affected by their occupational lifting status would be more likely to be excluded than the ones who had been least affected, and this would bias the estimation toward unity.

If we do not exclude participants who are treated for hypertension then the potential effects of occupational lifting on BP may be concealed, reversed, or otherwise distorted by effects from antihypertensive drugs and other types of heart medications.

It was the above mentioned problems that made us refrain from linear regression in the primary analyses. We recognize, however, that a conservative estimation of the effect of heavy occupational lifting on resting SBP in a linear regression model may provide meaningful information if the bias is taken into account in the interpretation of the results. We will therefore conduct a secondary analysis, in which the association between heavy occupational lifting and SBP will be investigated, first cross-sectionally and then prospectively (change in SBP [mm Hg] from examination n to examination n+1), by use of linear regression. Observations from participants who are treated with antihypertensive drugs or other types of heart medications will be excluded from an analysis similar to the primary analysis and performed both cross-sectionally and prospectively.

Generalized estimating equations will be used to estimate the parameters. Observations from the same person will be treated as repeated measurements. A first-order autoregressive correlation structure is assumed. Should the estimated covariance matrix fail to converge then we will resort to a variance component correlation structure. The expected difference between the exposed and the nonexposed will be estimated and presented with a 95% CI, based on the empiric SE.

#### Analysis on Other Types of Blood Pressure Measurements

It is presently not known if and how a person’s resting BP is influenced by occupational lifting activities. It is therefore of interest to also regard potential effects of occupational lifting on mean arterial pressure, DBP, and pulse pressure. For this reason, we will repeat the linear regression analyses described above on each of these outcomes. Furthermore, a prospective analysis will be applied where the outcome will be classified as a DBP case, similar to the analysis aforementioned relating occupational lifting to the risk of becoming an SBP case. The DBP case will be defined by the shift from no use of antihypertensive drugs in examination n to use of antihypertensive drugs in examination n+1 or an above median delta value of DBP (DBP in examination n+1−DBP in examination n).

#### Sensitivity to Choice of Comparison Group

According to our primary assessment of exposure, the exposed group would consist of participants whose work entailed heavy occupational lifting combined with walking, some handling of material, or heavy manual work. The comparison group would consist of the rest of the occupationally active participants, regardless of their type of occupational activity. We want to know how sensitive our analyses are to the choice of comparison group after adjustment for the included covariates. To shed some light on this issue, we plan to perform an additional set of linear regressions on SBP. In these particular analyses, we will split the comparison group into three different subgroups and thereby create an exposure variable with 4 instead of 2 categories. The statistical models, covariates, and inclusion criteria will otherwise be the same as they are in our previously defined linear regression analyses. The results will be presented as outlined in Table 6.

#### Sensitivity to the Definition of Hypertension

In our primary cross-sectional analysis, we will define hypertension as the use of antihypertensive drugs or a measured consultation SBP ≥140 mm Hg or DBP ≥90 mm Hg [25]. We recognize, however, that the cut-points could have been defined differently, eg, SBP ≥160 mm Hg or DBP ≥100 mm Hg [25]; SBP ≥180 mm Hg or DBP ≥110 mm Hg [25,26]; and SBP ≥130 mm Hg or DBP ≥80 mm Hg [27].

**Table 6:** Dummy table for the reporting of results of linear regressions on systolic blood pressure (SBP) as a function of occupational physical activity.

Occupational physical activity	Cross-sectional differences in SBP			Prospective differences in delta SBP		
	N^a^	Diff^b^	95% CI	N	Diff	95% CI
Heavy lifting		Ref^c^	—		Ref	—
Walking, some handling of material or heavy manual work but no heavy lifting						
Sitting or standing, some walking						
Predominantly sedentary work						

^a^Number of observations.

^b^Difference in mm Hg.

^c^Reference group.

We want to know whether the OR for hypertension as a function of heavy occupational lifting is sensitive to the definition of hypertension. We will therefore conduct two additional cross-sectional logistic regression analyses, which will be performed in the same way as the primary cross-sectional analysis but with the cut-points SBP ≥160 mm Hg or DBP ≥100 mm Hg and SBP ≥130 mm Hg or DBP ≥80 mm Hg instead of the traditional SBP ≥140 mm Hg or DBP ≥90 mm Hg.

#### Stratification by Age

A potential effect of occupational exposures might be more pronounced among people who are likely to be occupationally active throughout the approximately 10-year period that passes between the baseline and follow-up examinations than it is among people who have fulfilled the requirements for old-age pension (65 years of age) or early retirement (60 years of age) at the time of the follow-up examination. It is therefore possible that this study is more relevant among participants who are younger than 50 years at baseline than it is among those who are 50 years or older. For this reason, we will perform a sensitivity analysis in which the sample is stratified by age at baseline (≥ vs <50 years). The outcome, statistical model, inclusion criteria, and covariates will otherwise be the same as they were in the primary prospective analysis.

#### Linear Regression on Systolic Blood Pressure Without Exclusion of Participants Treated With Antihypertensive Drugs

As previously mentioned, we believe that any potential effect of occupational lifting on SBP may be concealed, reversed, or otherwise distorted by effects from antihypertensive drugs and other types of heart medications. It is, however, relevant to investigate the effect of the decision to exclude participants who were treated for antihypertensive drugs from sensitivity analysis 1 and, therefore, we will repeat the steps of that analysis, without the exclusion of medically treated participants.

### Substudy on Cardiac Damage

Data from the fourth and fifth examinations of the Copenhagen City Heart Study will be included for the cross-sectional and long-term associations between heavy occupational lifting and cardiac damage in a nested design. Early subclinical structural changes of the heart will be recognized by advanced echocardiographic analyses. We will compare participants exposed to heavy occupational lifting (cases) with matched participants who are not exposed to heavy occupational lifting (controls) both in the cross-sectional and longitudinal study. Controls will be matched on age and sex. Echocardiographic assessment will focus on early subclinical changes in cardiac structure primarily assessed by cardiac mass, indices of diastolic function, and global strain assessments. Analyses will be adjusted for confounders, including hypertension, diabetes, and BMI. With 200 exposed and 200 unexposed participants included in the echocardiographic analyses, we will have 80% power to detect a between-group difference of 1 in global longitudinal strain (equal to 5% difference based on an expected mean of 20) with a significance level (alpha) of 1.25%. The choice of alpha is adjusted to allow for comparison over several parameters of subclinical structural changes.

## Results

### Flow of Participants

**Figure 3:**
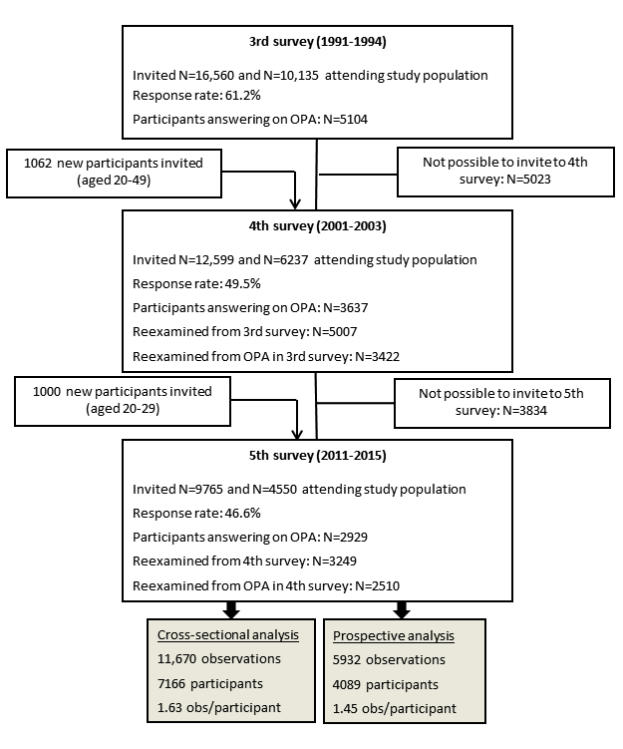
Flow of the observations and participants in the third, fourth, and fifth examinations of the Copenhagen City Heart Study. OPA: occupational physical activity; obs: observation.

In the third examination in 1991-1994, 10,135 out of 16,560 (61.20%) participants attended; in the fourth examination in 2001-2003, 6237 out of 12,599 (49.50%) participants attended; and in the fifth examination in 2011-2015, 4550 out of 9765 (46.59%) participants attended. On the basis of the inclusion criteria of responding to the level of OPA, 5031 observations were excluded from examination 3; 2600 from examination 4; and 1621 from examination 5. Hence, the final populations for the cross-sectional and prospective analysis are assumed to include less than 7166 participants in the cross-sectional analysis and less than 1850 participants in the prospective analysis (Figure 3), due to the additional inclusion criteria of measured BP and use of antihypertensive drugs. The information on BP and use of antihypertensive drugs will be provided after submission of this protocol paper.

### Descriptive Information of the Included Population

The population which will be included in the analysis will be set by the criteria for inclusion, described previously. Therefore, it is assumed that fewer participants will be included in the analysis than the amount of participants answering on the level of OPA, described in Tables 7 and 8.

### Differences in the Study Population

Smaller numerical differences were observed between the participants answering on the level of OPA and the attending participants.

Cross-sectionally, the participants responding to the level of OPA were 9.8 years younger (mean age 49.0 years among the participants answering on the level of OPA and 58.8 years among the attending), had a higher level of education than the attending participants (13.87% [1619/11,670] participants responding to the level of OPA were noneducated and 20.69% [4328/20,922] among the attending), and a higher proportion of the participants responding to the level of OPA stated to be exposed to heavy occupational lifting (14.04% [1638/11,670] among the participants responding to the level of OPA and 8.48% [1774/20,922] among the attending participants).

**Table 7:** Baseline characteristics of the participants responding to the level of occupational physical activity for the cross-sectional analysis; 11,670 observations on 7166 participants.

Cross-sectional analysis		Mean (SD)	n (%)	Range
Age (years)		49.0 (13.8)		20.3-99.7
Sex (% female)			5330 (54.3)	
Body mass index (kg/m^2^)		25.2 (4.3)		12.8-56.6
Smoking (% current smokers)			4292 (36.9)	
**Education**				
	Uneducated		1619 (14.1)	
	Low educated, <3 years		1806 (15.7)	
	Vocational education, 1-3 years		3123 (27.2)	
	Higher education, >3 years		2103 (18.3)	
	Academic education		2837 (24.7)	
**Occupational physical activity**				
	Predominantly sedentary		4407 (37.8)	
	Sitting or standing, some walking		4079 (35.0)	
	Walking, some handling of material		2729 (23.4)	
	Heavy manual work		455 (3.9)	
Occupational heavy lifting (% yes)			1830 (15.7)	
Vital exhaustion (sum, 0-17)		3.0 (3.5)		0-17
Cardiorespiratory fitness (% similar level as peers)			6390 (54.8)	
Observation per participant		2.0 (0.8)		1-3

Prospectively, the participants answering on the level of OPA were 11.0 years younger (mean age 48.0 years among participants responding to the level of OPA and 59.0 years among the attending). The smokers were 2.31 percentage points higher (38.96% [2311/5932] of the participants responding to the level of OPA were current smokers and 36.65% [6930/18,908] among the attending); they had a higher level of education than the attending participants (12.39% [735/5932] of the participants responding to the level of OPA were noneducated and 19.76% [3737/18,908] among the attending). A higher proportion of the participants responding to the level of OPA stated to be exposed to heavy occupational lifting (17.52% [1039/5932] among the participants responding to the level of OPA and 8.34% [1576/18,908] among the attending participants), and a higher proportion of the participants responding to the level of OPA stated to have a level of cardiorespiratory fitness similar to their peers (57.67% [3421/5932] among the participants responding to the level of OPA and 45.57% [8617/18,908] among the attending).

These nonsignificant differences between the attending participants and participants responding to the level of OPA in the cross-sectional and prospective populations may affect the prevalence of hypertension. The younger age of the participants responding to the level of OPA as well as their higher proportion of being educated might lower the prevalence of hypertension among these participants compared with those attending [28]. Conversely, may those participants responding to the level of OPA have a higher prevalence of hypertension due to their higher exposure to heavy occupational lifting than among the attending participants. In the prospective population, the small difference in proportion of participants stating to have a level of cardiorespiratory fitness similar to their peers, is not believed to affect the prevalence of hypertension, as the proportion of participants stating to have a higher level of cardiorespiratory fitness than their peers is similar among those participants responding to the level of OPA and those attending.

**Table 8:** Baseline characteristics of the participants responding to the level of occupational physical activity for the prospective analysis; 5932 observations on 4089 participants.

Prospective analysis		Mean (SD)	n (%)	Range
Age (years)		48.0 (11.8)		20.3-84.3
Sex (% female)			3301 (55.7)	
BMI (kg/m^2^)		25.0 (4.0)		16.0-52.5
Smoking (% current smokers)			2311 (39.1)	
**Education**				
	Uneducated		735 (12.6)	
	Low educated, <3 years		2142 (36.7)	
	Vocational education, 1-3 years		1983 (34.0)	
	Higher education, >3 years		380 (6.5)	
	Academic education		590 (10.1)	
**Occupational physical activity**				
	Predominantly sedentary		2113 (35.6)	
	Sitting or standing, some walking		2086 (35.2)	
	Walking, some handling of material		1497(25.2)	
	Heavy manual work		234 (3.9)	
Occupational heavy lifting (% yes)			1039 (17.5)	
Vital exhaustion (sum, 0-17)		2.9 (3.3)		0-17
Cardiorespiratory fitness (% similar level as peers)			3421 (57.7)	
Observation per participant		1.5 (0.5)		1-2

## Discussion

### Study Protocol

This study aims to contribute to the knowledge of risk for hypertension from heavy occupational lifting, and possibly thereby contribute to the prevention of cardiovascular disease by giving recommendations for participants exposed to heavy occupational lifting.

### Methodological Challenges

In the primary prospective analysis, the power would be insufficient if the outcome had been defined as hypertensive (yes or no). Therefore, we chose a case definition which included both hypertension and an above median increase in SBP of the study population from examination n to n+1. The proposed analyses have some limitations, such as the self-reported exposure to occupational lifting and level of cardiorespiratory fitness. Previous studies show that self-reported exposure to occupational lifting may be affected by recall bias [29,30]. Also the collection of BP only in consultation during rest is a limitation due to the lower prognostic value than obtained by monitoring of 24 hours BP or BP during sleep [31,32]. Furthermore, a previous study has shown occupational lifting to reduce the odds for having prolonged working hours [33]; however, this is not possible to adjust for in this analysis due to the lack of information on amount of weekly working hours. It could also be speculated that the range and variety of the exposure to occupational lifting could be limited due to the Danish Working Environment Authority guideline for occupational lifting [34], stating that carrying, lifting, pulling, and pushing of nonliving burdens below 3 kg are not classified as heavy lifting, and workers should not lift or carry burdens heavier than 20 kg.

Some of the strengths in the proposed analysis are the follow-up time of 8 to 10 years and the determination of hypertension based both on the use of prescription medicine and the resting BP in mm Hg. This limits the risk of classifying a participant as false negative (eg, using antihypertensives and therefore having a resting BP below the threshold). Another strength is the randomly selected study population.

### Implications of the Proposed Analysis

Since one-third of the workforce in Europe reports to carry or move heavy loads regularly during working hours (6th survey in Eurofound) and hypertension is a major risk factor for cardiovascular disease and mortality [1;2], a positive association between occupational lifting and risk for hypertension could reveal a potential for improved prevention for hypertension by reducing exposure to occupational lifting in the population. This could, for example, be achieved by using technical lifting devices and automatization of manual work tasks currently requiring heavy lifting. This is particularly the case because a positive association could be considered as a reflection of a physiological mechanism and therefore must be assumed to apply for the majority of humans exposed to occupational lifting. Conversely, a negative association would not be assumed as a reflection of a physiological mechanism before the negative association had been verified in populations not subject to restrictive regulations of occupational lifting, as employees in Denmark are. Moreover, a null finding would also propose a need for additional investigations of this association in populations with wider ranges of exposure to occupational lifting. Since these proposed analyses will be applied to a randomly selected adult population and is planned to be verified in another randomly selected adult Danish population, these results may be generalized to the Danish adult population engaged in work including occupational lifting.

## Abbreviations

BMI: body mass index
BP: blood pressure
DBP: diastolic blood pressure
IHD: ischemic heart disease
OPA: occupational physical activity
OR: odds ratio
SBP: systolic blood pressure

## References

[1] Olsen MH, Angell SY, Asma S, Boutouyrie P, Burger D, Chirinos JA, Damasceno A, Delles C, Gimenez-Roqueplo A, Hering D, López-Jaramillo P, Martinez F, Perkovic V, Rietzschel ER, Schillaci G, Schutte AE, Scuteri A, Sharman JE, Wachtell K, Wang JG (2016). A call to action and a lifecourse strategy to address the global burden of raised blood pressure on current and future generations: the Lancet Commission on hypertension. Lancet.

[2] Antikainen RL, Moltchanov VA, Chukwuma C, Kuulasmaa KA, Marques-Vidal PM, Sans S, Wilhelmsen L, Tuomilehto JO, WHO MONICA Project (2006). Trends in the prevalence, awareness, treatment and control of hypertension: the WHO MONICA Project. Eur J Cardiovasc Prev Rehabil.

[3] Fransson E, De Faire U, Ahlbom A, Reuterwall C, Hallqvist J, Alfredsson L (2004). The risk of acute myocardial infarction: interactions of types of physical activity. Epidemiology.

[4] Petersen CB, Eriksen L, Tolstrup JS, Søgaard K, Grønbaek M, Holtermann A (2012). Occupational heavy lifting and risk of ischemic heart disease and all-cause mortality. BMC Public Health.

[5] MacDougall JD, Tuxen D, Sale DG, Moroz JR, Sutton JR (1985). Arterial blood pressure response to heavy resistance exercise. J Appl Physiol (1985).

[6] Clays E, De Bacquer D, Van Herck K, De Backer G, Kittel F, Holtermann A (2012). Occupational and leisure time physical activity in contrasting relation to ambulatory blood pressure. BMC Public Health.

[7] Hannerz H, Holtermann A (2016). Ischaemic heart disease among workers in occupations associated with heavy lifting. Int J Occup Med Environ Health.

[8] Williams M, Haskell W, Ades P, Amsterdam E, Bittner V, Franklin B (2007). Resistance exercise in individuals with and without cardiovascular disease update: a scientific statement from the American Heart Association Council on Clinical Cardiology and Council on Nutrition, Physical Activity, and Metabolism. Circulation.

[9] Cornelissen V, Fagard R, Coeckelberghs E, Vanhees L (2011). Impact of resistance training on blood pressure and other cardiovascular risk factors: a meta-analysis of randomized, controlled trials. Hypertension.

[10] (2014). The National Research Centre for the Working Environment.

[11] Schnohr P (2009). Physical activity in leisure time: impact on mortality. Risks and benefits. Dan Med Bull.

[12] Landis JR, Koch GG (1977). The measurement of observer agreement for categorical data. Biometrics.

[13] Lewington S, Clarke R, Qizilbash N, Peto R, Collins R (2002). Age-specific relevance of usual blood pressure to vascular mortality: a meta-analysis of individual data for one million adults in 61 prospective studies. Lancet.

[14] Doumas M, Papademetriou V, Faselis C, Kokkinos P (2013). Gender differences in hypertension: myths and reality. Curr Hypertens Rep.

[15] Quan H, Chen G, Walker RL, Wielgosz A, Dai S, Tu K, Campbell NRC, Hemmelgarn BR, Hill MD, Johansen H, McAlister FA, Khan N, Hypertension Outcome and Surveillance Team (2013). Incidence, cardiovascular complications and mortality of hypertension by sex and ethnicity. Heart.

[16] Kronborg CN, Hallas J, Jacobsen IA (2009). Prevalence, awareness, and control of arterial hypertension in Denmark. J Am Soc Hypertens.

[17] Papathanasiou G, Zerva E, Zacharis I, Papandreou M, Papageorgiou E, Tzima C, Georgakopoulos D, Evangelou A (2015). Association of high blood pressure with body mass index, smoking and physical activity in healthy young adults. Open Cardiovasc Med J.

[18] Chen J, Das S, Barlow CE, Grundy S, Lakoski SG (2010). Fitness, fatness, and systolic blood pressure: data from the Cooper Center Longitudinal Study. Am Heart J.

[19] Saladini F, Benetti E, Fania C, Mos L, Casiglia E, Palatini P (2016). Effects of smoking on central blood pressure and pressure amplification in hypertension of the young. Vasc Med.

[20] Dochi M, Sakata K, Oishi M, Tanaka K, Kobayashi E, Suwazono Y (2009). Smoking as an independent risk factor for hypertension: a 14-year longitudinal study in male Japanese workers. Tohoku J Exp Med.

[21] Tang K, Rashid R, Godley J, Ghali W (2016). Association between subjective social status and cardiovascular disease and cardiovascular risk factors: a systematic review and meta-analysis. BMJ Open.

[22] Appels A (1990). Mental precursors of myocardial infarction. Br J Psychiatry.

[23] Prescott E, Holst C, Grønbaek M, Schnohr P, Jensen G, Barefoot J (2003). Vital exhaustion as a risk factor for ischaemic heart disease and all-cause mortality in a community sample. A prospective study of 4084 men and 5479 women in the Copenhagen City Heart Study. Int J Epidemiol.

[24] Cornelissen V, Smart N (2013). Exercise training for blood pressure: a systematic review and meta-analysis. J Am Heart Assoc.

[25] Mancia G, Fagard R, Narkiewicz K, Redon J, Zanchetti A, Böhm M, Christiaens T, Cifkova R, De Backer G, Dominiczak A, Galderisi M, Grobbee DE, Jaarsma T, Kirchhof P, Kjeldsen SE, Laurent S, Manolis AJ, Nilsson PM, Ruilope LM, Schmieder RE, Sirnes PA, Sleight P, Viigimaa M, Waeber B, Zannad F, Task Force for the Management of Arterial Hypertension of the European Society of Hypertension and the European Society of Cardiology (2014). 2013 ESH/ESC practice guidelines for the management of arterial hypertension. Blood Press.

[26] Leung AA, Nerenberg K, Daskalopoulou SS, McBrien K, Zarnke KB, Dasgupta K, Cloutier L, Gelfer M, Lamarre-Cliche M, Milot A, Bolli P, Tremblay G, McLean D, Tobe SW, Ruzicka M, Burns KD, Vallée M, Prasad GV, Lebel M, Feldman RD, Selby P, Pipe A, Schiffrin EL, McFarlane PA, Oh P, Hegele RA, Khara M, Wilson TW, Penner SB, Burgess E, Herman RJ, Bacon SL, Rabkin SW, Gilbert RE, Campbell TS, Grover S, Honos G, Lindsay P, Hill MD, Coutts SB, Gubitz G, Campbell NRC, Moe GW, Howlett JG, Boulanger J, Prebtani A, Larochelle P, Leiter LA, Jones C, Ogilvie RI, Woo V, Kaczorowski J, Trudeau L, Petrella RJ, Hiremath S, Drouin D, Lavoie KL, Hamet P, Fodor G, Grégoire JC, Lewanczuk R, Dresser GK, Sharma M, Reid D, Lear SA, Moullec G, Gupta M, Magee LA, Logan AG, Harris KC, Dionne J, Fournier A, Benoit G, Feber J, Poirier L, Padwal RS, Rabi DM, CHEP Guidelines Task Force (2016). Hypertension Canada's 2016 Canadian Hypertension Education Program guidelines for blood pressure measurement, diagnosis, assessment of risk, prevention, and treatment of hypertension. Can J Cardiol.

[27] Whelton P, Carey R, Aronow W, Casey Jr DJ, Collins K, Dennison H, DePalma SM, Gidding S, Jamerson KA, Jones DW, MacLaughlin EJ, Muntner P, Ovbiagele B, Smith Jr SC, Spencer CC, Stafford RS, Taler SJ, Thomas RJ, Williams Sr KA, Williamson JD, Wright Jr JT (2017). 2017 ACC/AHA/AAPA/ABC/ACPM/AGS/APhA/ASH/ASPC/NMA/PCNA Guideline for the prevention, detection, evaluation, and management of high blood pressure in adults: a report of the American College of Cardiology/American Heart Association Task Force on clinical practice guidelines. J Am Coll Cardiol.

[28] Wen W, Luo R, Tang X, Tang L, Huang HX, Wen X, Hu S, Peng B (2015). Age-related progression of arterial stiffness and its elevated positive association with blood pressure in healthy people. Atherosclerosis.

[29] Fransson E, Knutsson A, Westerholm P, Alfredsson L (2008). Indications of recall bias found in a retrospective study of physical activity and myocardial infarction. J Clin Epidemiol.

[30] Stock SR, Fernandes R, Delisle A, Vézina N (2005). Reproducibility and validity of workers' self-reports of physical work demands. Scand J Work Environ Health.

[31] Hansen TW, Thijs L, Li Y, Boggia J, Kikuya M, Björklund-Bodegård K, Richart T, Ohkubo T, Jeppesen J, Torp-Pedersen C, Dolan E, Kuznetsova T, Stolarz-Skrzypek K, Tikhonoff V, Malyutina S, Casiglia E, Nikitin Y, Lind L, Sandoya E, Kawecka-Jaszcz K, Imai Y, Wang J, Ibsen H, O'Brien E, Staessen JA, International Database on Ambulatory Blood Pressure in Relation to Cardiovascular Outcomes Investigators (2010). Prognostic value of reading-to-reading blood pressure variability over 24 hours in 8938 subjects from 11 populations. Hypertension.

[32] Hermida RC, Ayala DE, Fernández JR, Mojón A (2013). Sleep-time blood pressure: prognostic value and relevance as a therapeutic target for cardiovascular risk reduction. Chronobiol Int.

[33] Hannerz H, Holtermann A (2014). Heavy lifting at work and risk of ischemic heart disease: protocol for a register-based prospective cohort study. JMIR Res Protoc.

[34] Danish Working Environment Authority.

